# Biologic Treatments in Interstitial Lung Diseases

**DOI:** 10.3389/fmed.2019.00041

**Published:** 2019-03-13

**Authors:** Theodoros Karampitsakos, Argyro Vraka, Demosthenes Bouros, Stamatis-Nick Liossis, Argyris Tzouvelekis

**Affiliations:** ^1^5th Department of Pneumonology, General Hospital for Thoracic Diseases Sotiria, Athens, Greece; ^2^First Academic Department of Pneumonology, Hospital for Thoracic Diseases, Sotiria Medical School, National and Kapodistrian University of Athens, Athens, Greece; ^3^Division of Rheumatology, Department of Internal Medicine, Patras University Hospital, University of Patras Medical School, Patras, Greece

**Keywords:** interstitial lung diseases, biologic treatments, pulmonary fibrosis, treatment, safety

## Abstract

Interstitial lung diseases (ILD) represent a group of heterogeneous parenchymal lung disorders with complex pathophysiology, characterized by different clinical and radiological patterns, ultimately leading to pulmonary fibrosis. A considerable proportion of these disease entities present with no effective treatment, as current therapeutic regimens only slow down disease progression, thus leaving patients, at best case, with considerable functional disability. Biologic therapies have emerged and are being investigated in patients with different forms of ILD. Unfortunately, their safety profile has raised many concerns, as evidence shows that they might cause or exacerbate ILD status in a subgroup of patients. This review article aims to summarize the current state of knowledge on their role in patients with ILD and highlight future perspectives.

## Introduction

Interstitial lung diseases (ILD) are a group of heterogeneous parenchymal lung disorders, characterized by different clinical and radiological patterns (1, 2). Despite an exponential increase in our knowledge and the advent of novel therapies, treatment remains ineffective for a considerable proportion of patients (3–13). Biologic treatments comprise a wide group of compounds with natural origin produced by biotechnology and other cutting-edge technologies (14); yet, this term mainly refers to the subgroup of complex molecules representing targeted therapy, such as monoclonal antibodies and receptor fusion proteins (15). The last years have seen the emergence of biologic treatments for the treatment of several immune and oncologic disorders (16–18). The most extensively used are tumor necrosis factor-α (TNF-a) inhibitors, B-cell-targeted therapies, T cell co-stimulatory molecule blockers, and immune check point inhibitors. With regards to ILDs, there is established knowledge on the use of biologic therapies in patients with connective tissue disorders (CTD-ILDs) and sarcoidosis (12, 16, 19–21). Despite old skepticism (7, 22–27), there has been recently a shift toward targeting the immune system as a therapeutic option for different forms of interstitial lung inflammation and fibrosis (9, 28–33). Unfortunately, their safety profile has raised many concerns, as evidence shows that they might exacerbate or cause *de novo* development of ILD in a subgroup of patients (34–36) (Table 1). This review article aims to summarize the current state of knowledge on their role in patients with ILD and highlight future perspectives.

**Table 1:** Lung toxicity of biologic treatments.

**Biologic treatment**	**Radiologic findings**	**References**
Anti-TNFα	Aseptic granulomatous pulmonary nodules Interstitial lung infiltrates Incidence of DI-ILD:0.5–3%	(37–40)
Rituximab	Organizing Pneumonia ARDS	(41)
Tocilizumab	Organizing Pneumonia Exacerbation of ILD Pneumonitis	(42–44)
Abatacept	Rarely causes or exacerbates ILD	(45)

*ARDS, Acute Respiratory Distress Syndrome; DI-ILD, Drug Induced- Interstitial Lung Disease; TNF, Tumor Necrosis Factor*.

## Sarcoidosis (Table 2)

Prednisolone remains the cornerstone of sarcoidosis treatment (55). Biologic therapies currently represent a fruitful therapeutic alternative in sarcoidosis cases refractory to first line immunomodulatory agents including corticosteroids, methotrexate, azathioprine, leflunomide and mycophenolate mofetil (56). TNFα inhibitors in combination with low dose prednisolone or methotrexate have been suggested in: (i) chronic progressive pulmonary disease, (ii) debilitation by lupus pernio, (iii) persistent neurosarcoidosis, (iv) persistent cardiac sarcoidosis (55). Infliximab has shown superior response rates in pulmonary sarcoidosis compared to etanercept and adalimumab (46, 47, 50, 51, 57). In particular, a randomized controlled trial (RCT) enrolling 148 patients with chronic pulmonary sarcoidosis showed that infliximab led to a statistically significant 2.5% improvement in forced vital capacity (FVC%pred) after 24 weeks of treatment (46). Results from other non-randomized trials were rather conflicting (47, 48). Unfortunately, almost 2/3 of patients with sarcoidosis receiving infliximab demonstrated relapse following drug-cessation (49). Adalimumab has shown acceptable tolerability and efficacy profile as indicated by improvements in FVC% pred, 6 Minute-Walk-Distance (6MWD) and Borg scale over a period of 52 weeks in a small cohort of patients with refractory pulmonary sarcoidosis (50). A phase 2 trial of etanercept in patients with pulmonary sarcoidosis was prematurely terminated due to unfavorable outcomes (51). Furthermore, golimumab (TNFα inhibitor) and ustekinumab (a monoclonal antibody targeting both IL-12 and IL-23) failed to show efficacy in patients with pulmonary and/or cutaneous sarcoidosis in an RCT with 173 patients (52). Finally, rituximab had an acceptable safety profile but inconsistent efficacy in a small cohort of patients with different genetic backgrounds and refractory pulmonary sarcoidosis; thus, its use through a personalized medicine approach could be viable in the future (53).

**Table 2:** Biologic treatments in pulmonary sarcoidosis.

**Study**	**Biologic agent**	**Mechanism of action**	**Number of patients/Outcome**	**References**
Baughman et al.	Infliximab	Chimeric monoclonal antibody against TNF	148 patients Improvement of 2.5% in FVC over 24 weeks	(46)
Rossman et al.	Infliximab	Chimeric monoclonal antibody against TNF	19 patients No significant improvement over 6 and 14 weeks	(47)
Vorselaars et al.	Infliximab	Chimeric monoclonal antibody against TNF	56 patients Improvement of 6.6% in FVC Uptake value on ^18^F-FDG-PET predictive of response	(48)
Vorselaars et al.	Infliximab	Chimeric monoclonal antibody against TNF	47 patients Relapse 62% Increased SUV, IL-2r predictors	(49)
Sweiss et al.	Adalimumab	Humanized monoclonal antibody against TNF	11 patients Improvement in FVC (4), stabilization in FVC (7), improvement in 6MWD (5), improvement in Borg (9) over 24/52 weeks	(50)
Utz et al.	Etanercept	Receptor antagonist of TNF	17 patients Excessive treatment failure	(51)
Judson et al.	Ustekinumab/ golimumab	Humanized monoclonal antibody against IL12,IL23/and against TNF, respectively	173 patients (pulmonary or cutaneous) No significant improvement over 28 weeks	(52)
Sweiss et al.	Rituximab	Humanized monoclonal antibody against CD20	10 patients >5% improvement in FVC (5) improvement by >30 m in 6MWD (5) over 24/52 weeks	(53)
NCT02888080	Canakinumab	Human monoclonal antibody against IL-1 b	Change in PFTs from baseline to week 24/Recruiting	(54)

*CD, Cluster of Differentiation; IL, interleukin; ^18^F-FDG-PET, Fludeoxyglucose (^18^F) Positron Emission Tomography; FVC, Forced Vital Capacity; PFTs, Pulmonary Function Tests; SUV, Standardized Uptake Value; TNF, Tumor Necrosis Factor; 6MWD, 6 Minute Walk Distance*.

Elevated C-reactive protein (CRP) levels and TNFα Gly308Ala polymorphisms have been found to be predictive of response to anti-TNFα therapy, while soluble IL-2 receptor serum levels ≥4,000 pg·mL^−1^ at start of therapy were predictive of relapse (49, 58). Moreover, ^18^8F-FDG-PET showed remarkable predictive accuracy in identifying patients that responded or relapsed following infliximab treatment (48, 49).

A broad spectrum of adverse events have been associated with the use of TNF-α inhibitors including anaphylactic reactions, reactivation of latent infections, neurological (i.e., demyelinating diseases) and autoimmune disorders and maybe in some cases malignancy (55, 59, 60). The paradoxical response, denominated sarcoid-like granulomatosis, has also been reported (61).

In conclusion, current evidence based on expert opinion suggests the use of biologic treatments in severe refractory pulmonary sarcoidosis. TNFα-inhibitors are preferred for patients with persistent disease despite treatment with corticosteroids and other second-line immunomodulatory compounds, especially in cases of life-threatening disease. However, such strategies need thorough pre-treatment evaluation and multidisciplinary approaches (12).

## Idiopathic Pulmonary Fibrosis (Figure 1, Table 3)

The treatment of IPF has been revolutionized by the advent of two novel compounds, pirfenidone and nintedanib (3–11). Nevertheless, both compounds only slow down disease progression; thus, at best leave patients with considerable functional disability. Therefore, the need for alternative therapeutic options remains amenable (75–78).

**Figure 1:**
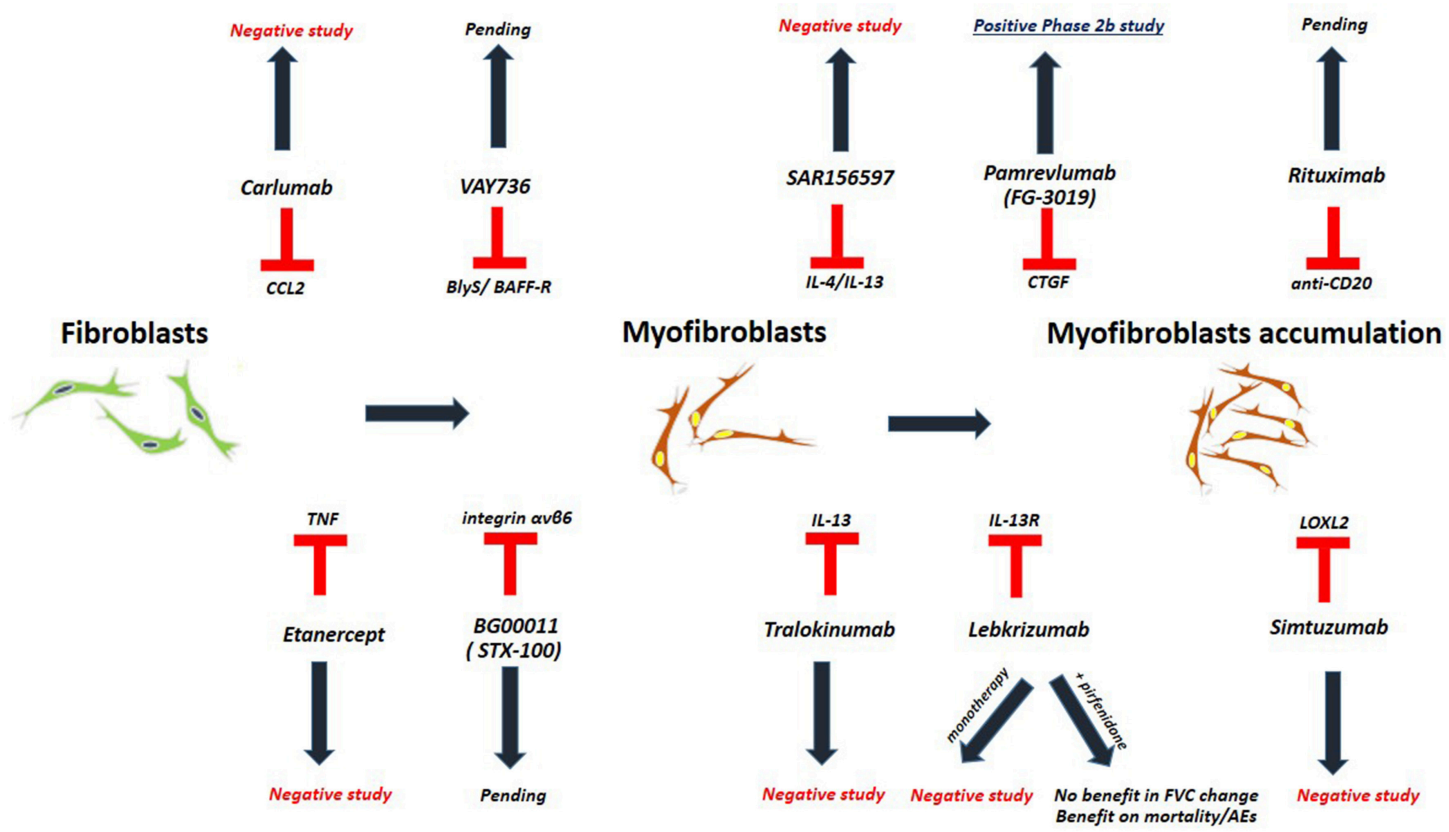
Studies investigating biologic treatments in patients with IPF.

**Table 3:** Phase 2 clinical trials for biologic treatments in patients with IPF.

**Biologic agent**	**Mechanism of action**	**Outcome**	**References**
Carlumab	CCL2 inhibitor	Negative study	NCT00786201 (62)
Etanercept	Receptor antagonist of TNF	Negative study	NCT00063869 (63)
Imatinib	Tyrosine kinase inhibitor	Negative study	NCT00131274 (64)
Lebrikizumab	anti- IL13	Monotherapy: Negative study Combination with pirfenidone: Trend for benefit on AE/mortality	NCT01872689 (65–67)
Pamrevlumab (FG-3019)	Monoclonal antibody against CTGF	Positive phase 2 open label trial	NCT01262001 (68)
simtuzumab	Anti-LOXL2	Negative study	NCT01769196 (69)
Tralokinumab	Anti-IL13	Negative study	NCT01629667 (70)
BG00011 (STX-100)	Humanized monoclonal antibody against integrin αvβ6	Pending	NCT01371305 (66)
VAY736	Monoclonal antibody against BlyS/ BAFF-R	Pending	NCT03287414 (71)
SAR156597	Bispecific monoclonal antibody against IL-4 and IL-13	Negative study	NCT02921971 (72)
Rituximab	anti-CD20	Pending	NCT01969409 NCT03286556 (73, 74)

*BAFF-R, B cell activating factor; CCL2, chemokine (C-C motif) ligand 2; CTGF, Connective Tissue Growth Factor; IL, interleukin; LOXL2, Lysyl oxidase homolog 2; RCT, Randomized Controlled Trial; TNF, Tumor Necrosis Factor*.

Biologic agents represent one such option, yet with disappointing results. The clinical trial of carlumab, a monoclonal antibody against CC-chemokine ligand 2 (CCL2), was stopped prematurely as patients in the carlumab-treatment-arm experienced greater functional decline compared to the patients in the placebo-treatment-arm (62). TNFa-blocking agents such as etanercept showed no efficacy in patients with IPF (63). Imatinib, a tyrosine kinase inhibitor with multiple biologic properties, did not affect survival or lung function of patients with IPF (64). The study of simtuzumab, a monoclonal antibody against lysyl oxidase-like 2 (LOXL2), was also a negative study (69). Most recently, two anti-IL-13 monoclonal antibodies have entered the pipeline of clinical trials for IPF. Tralokinumab had an acceptable safety and tolerability profile; yet, key efficacy endpoints were not met (70). Monotherapy with lebrikizumab, another anti-IL-13 monoclonal antibody, did not result in a benefit on lung function or mortality over 52 weeks (65). Combination of lebrikizumab and pirfenidone was well-tolerated but did not meet the primary endpoint of FVC% decline; yet, a trend toward beneficial effects on mortality and acute exacerbations was observed (66, 67). Furthermore, SAR156597, a monoclonal bispecific antibody targeting IL-4 and IL-13, failed to halt disease progression either as monotherapy or in combination with standard-of-care antifibrotics (72). A Phase 2 open label trial of pamrevlumab (FG-3019), a monoclonal antibody blocking the downstream effects of connective tissue growth factor (CTGF), showed an acceptable safety and efficacy profile and thus a phase III clinical trial is currently anticipated (68, 79, 80). Safety and efficacy of VAY736, a monoclonal antibody against the cytokine BlyS, a B cell activating factor, is also currently being tested in a phase 2 study (71). BG00011 (STX-100), a humanized monoclonal antibody against integrin αvβ6, demonstrated an acceptable safety profile and its efficacy is currently investigated in a phase 2b study (66, 81). Finally, rituximab ± intravenous immunoglobulin showed 1-year survival benefit in a small cohort of patients with IPF undergoing acute exacerbation compared to historical controls (82). A Phase 2 trial of rituximab in IPF aiming to reduce titers of autoantibodies to HEp-2 Cells over a 9-months period of follow up, has been recently completed (73, 83). In addition, the results of autoantibody reduction for acute exacerbations of IPF (STRIVE-IPF) are greatly anticipated (74).

## Connective Tissue Disease-Associated Interstitial Lung Disease (CTD-ILD) Rheumatoid Arthritis

Pulmonary complications represent an important extra-articular feature of rheumatoid arthritis and a major cause of mortality and worse quality of life (16). The decision to treat them requires a multidisciplinary approach weighting: (i) the disease severity and patients' clinical status, (ii) the potential benefits of early therapy (i.e., treatment of inflammation before fibrosis is established) and (iii) the risk of adverse events (i.e., immunosuppression especially for patients with established fibrosis or severe bronchiectatic lesions). Given the lack of consensus over clinical trials, management is currently based on expert opinion. The recent emergence of novel anti-fibrotic compounds for the IPF-UIP-lung holds promise for the RA-UIP-lung (84–87) and the first randomized trial of antifibrotics in RA-ILD (TRAIL trial) is currently under investigation (84). To this end, biologic treatments may present with beneficial outcomes in a proportion of patients with refractory RA-ILD.

Rituximab represents the most widely used biologic treatment in patients with rapidly progressive RA-ILD who are unresponsive to first line therapeutic compounds including corticosteroids and methotrexate (88). Unfortunately, evidence is based on small observational studies and thus further data is required (89–97). A recent prospective, observational cohort study enrolling 43 patients on rituximab and 309 patients on TNF-α inhibitors, demonstrated better long-term survival in patients receiving rituximab than in those receiving TNF-α inhibitor, as event rates were 53.0 and 94.8 per 1,000 person years, respectively (98).

The use of TNF-α inhibitors yielded controversial safety and efficacy results in patients with RA-ILD. Caveats following their use in CTD-ILD parallel those previously described in sarcoidosis. Despite their effectiveness in improving clinical status and slowing down articular disease progression, lung toxicity remains a major concern (99–103). Small case series of patients with RA-ILD have shown that infliximab and etanercept could improve dyspnea and cough, as well as stabilize disease functional status (104–107). On the other hand, safety concerns have been raised for current TNF-α inhibitors infliximab (108–111), etanercept (112–116), adalimumab (117–121), golimumab (90), and certolizumab (37, 122, 123) considering reports for ILD exacerbation. Importantly, TNF- induced ILD could be rapidly progressive and even fatal, especially in patients with preexisting ILD (34, 124–127). Nonetheless, large cohorts of patients with RA reported no association between anti-TNF agents and ILD development or progression (128, 129). Caution should be used for elderly patients, as they represent a high-risk and frail group of patients (100).

Data for other agents including abatacept, tocilizumab and anakinra are still scarce. Abatacept has shown an acceptable safety and efficacy profile, as assessed by dyspnea, functional indicators and radiological extent of inflammation, in both large RCTs (130) and smaller case studies (45, 90, 102, 131, 132). The use of tocilizumab yielded conflicting results and it seems to be beneficial only in a small subgroup of patients with RA-ILD (42, 90, 102, 126, 133–137). Isolated cases of ILD-exacerbation following treatment with tocilizumab have been described (138). Finally, anakinra, an IL-1 receptor antagonist, is rarely, if ever, employed, in the treatment of patients with RA-ILD (126, 139).

## Scleroderma

Until recently, the standard treatment for systemic sclerosis-associated ILD (SSc-ILD) was considered to be cyclophosphamide, based on the results of Scleroderma Lung Study (140). However, previously reported data from small-scale studies depicted beneficial effects of mycophenolate mofetil in SSc-ILD (141–143). The recently reported large-scale, randomized, double-blind Scleroderma Lung Study II comparing head-to-head cyclophosphamide vs. mycophenolate mofetil disclosed that mycophenolate mofetil was as effective as cyclophosphamide but with a better safety profile. Thus, mycophenolate mofetil has been established as the current standard of care for SSc-ILD (144). The statistically significant but clinically rather small benefit from the use of such treatment along with the commonly resistant nature of SSc-ILD, clearly underscores the need for novel treatments. Biologic agents, particularly rituximab, have been evaluated in small-scale studies in a minority of patients with progressive, treatment-resistant disease (145). The results of a multicenter, open label, comparative study evaluating rituximab on top of standard treatment (*n* = 33) vs. standard treatment alone (*n* = 18) showed that patients in the rituximab group had a 6% increase of FVC compared to baseline values at 2 years of treatment, a benefit that apparently was preserved later on; however, the number of patients at 7 years of treatment was too small for safe conclusions (146). Direct comparison between the rituximab group and the standard-treatment group disclosed a statistically significant benefit for the rituximab-treated patients. Other studies have reported results along the same lines (19, 20, 145, 147–149). Nevertheless, formal, multicenter, large-scale studies are clearly needed to evaluate the value of B-cell depletion treatment(s) in patients with SSc-ILD. A phase III trial evaluating the effects of the anti-IL-6 receptor monoclonal antibody tocilizumab was terminated despite relatively promising results in the earlier phase trials (150, 151) and the results from the use of belimumab, an anti-BLyS monoclonal antibody, have been evaluated only in one study with a small number of patients (*n* = 9) with clinically non-significant SSc-ILD (152).

## Myositis/ Antisynthetase Syndrome

ILDs represent a major cause of mortality in dermatomyositis (DM), polymyositis (PM) and antisynthetase syndrome. Most common antibodies in patients with myositis-ILD include anti-EJ, anti-PL12, anti-PL7, anti-Jo1, anti-OJ and anti-KS (153). Biologics have been used in cases of myositis-associated-ILD refractory to more commonly used immunomodulatory agents such as corticosteroids, azathioprine and mycophenolate mofetil (92, 153). Data derived from case series, case reports and retrospective studies suggested clinical, functional and radiologic benefits from rituximab in patients with progressive ILD associated with PM/DM/ antisynthetase syndrome (92, 154–161). Basiliximab, a monoclonal antibody blocking the alpha chain (CD25) of the IL-2 receptor complex, resulted in radiologic and functional improvement in three out of four cases of clinically amyopathic dermatomyositis (CADM) with anti-MDA5 positivity and rapidly progressive ILD (162). However, prior to the application of such therapies, exclusion of other causes of lung function deterioration such as drug-induced pneumonitis, superimposed infection and respiratory muscle weakness is mandatory.

## Future Perspectives and Concluding Remarks (Table 3)

ILDs represent disease paradigms of unknown pathogenesis, unpredictable clinical course and relatively ineffective therapeutic approaches. Biologic therapies may offer an effective alternative in progressive and refractory cases. Early identification of these patients is of paramount importance. Unfortunately, current physiologic biomarkers neither provide mechanistic insights in disease endotypes nor they predict disease clinical course. While ILDs are associated with several underlying mechanisms, currently applied regimens target specific pathways and thus there is still an amenable need for novel compounds. The development of biologics for the treatment of fibrotic lung diseases may hold promise considering the potential for disease modulation (163).

Biologic agents have shown to have a major impact in severe refractory cases of sarcoidosis. Furthermore, canakinumab, a human monoclonal antibody against IL-1 b, has entered the pipeline of clinical trials for sarcoidosis and the results are greatly anticipated (54). Unfortunately, the majority of biologic agents in IPF have, so far, led to disappointing results mainly due to the fact that they target immune-mediated inflammation and not fibrosis. Application of oncologic and personalized medicine approaches represent crucial steps toward successful implementation of biologic agents in lung fibrosis (164). The advent and implementation of high-throughput computational tools could identify biomarkers able to distinguish patients' endotypes and thus predict the subgroup of patients which are more likely to benefit from specific biologic interventions (165, 166). Biologic enrichment of future clinical trials and implementation of biomarkers as end-points could have a crucial impact toward this direction. Systematic pre-treatment assessment for latent infections and immunocompromise is mandatory prior treatment initiation to avoid undesirable adverse-events. Thoughtful monitoring and multi-disciplinary care with rheumatologists and pulmonologists are strongly encouraged.

## Author Contributions

TK and AV wrote the manuscript. The manuscript was significantly modified by DB, S-NL, and AT. All authors offered intellectual contribution.

### Conflict of Interest Statement

The authors declare that the research was conducted in the absence of any commercial or financial relationships that could be construed as a potential conflict of interest.
